# Quality of option B + prevention of mother-to-child transmission of HIV services in public hospitals of Gamo zone, Southern Ethiopia, mixed approach

**DOI:** 10.1186/s12887-023-03901-w

**Published:** 2023-03-02

**Authors:** Yosef Haile, Zeleke Gebru, Tesfaye Feleke, Yonas Fissha Adem

**Affiliations:** 1 Arba Minch University, College of Medicine and Health Sciences, School of Public Health, Arba Minch, Ethiopia; 2 Department of Public Health, Dessie College of Health Sciences, Dessie, Ethiopia

**Keywords:** PMTCT, Mother to a child, HIV AIDS, Transmission, Gamo zone

## Abstract

**Background:**

Prevention of mother-to-child transmission service is a comprehensive package of services planned to reduce the risk of mother-to-child transmission of HIV. It is very crucial to determine the level of quality of PMTCT services in this study area since other studies in our country omitted several variables in each category of the Donobedian model. Therefore, this study aimed to determine the level of quality of option B + PMTCT of HIV services.

**Methods:**

An institution-based cross-sectional study design with both quantitative and qualitative data collection method was employed. Donabedian’s model was used to assess the level of quality of PMTCT service. A total of 422 pregnant women were used to assess the level of satisfaction of clients. An inventory of resources and direct observation was done to assess the quality of the input and output component of the Donobedian model respectively. In addition to satisfaction items, 12 output-related items were also used to assess quality in the output dimension. Finally, those hospitals that scored above 90% in each component of the Donovedian model were categorized as having good quality. Finally, twelve in-depth interviews were conducted to explore barriers to the quality of option B + PMTCT services. The qualitative data were analyzed using the thematic analysis method and finally, it was presented with the quantitative result through triangulation.

**Results:**

No hospitals simultaneously met the requirements for good quality in all three dimensions of option B + PMTCT service quality. Only one hospital out of the four hospitals met the requirements for good quality of PMTCT service in the input dimension. Regarding the process and output dimension's quality of PMTCT services, two of the hospitals met the criteria for good quality. One hospital out of the total exhibited poor performance in all three dimensions of service quality for option B + PMTCT services.

**Conclusion:**

According to this study no hospitals simultaneously met the requirements for good quality in all three dimensions of option B + PMTCT service quality. PMTCT unit performance must be continuously monitored, reviewed, and supervised. To obtain the minimum required resources primary hospitals must be supported.

## Introduction

Vertical or mother-to-child transmission (MTCT) occurs when a mother who is HIV-positive passes the virus on to her child during pregnancy, labor, delivery, or breastfeeding [1]. HIV transmission rate ranges from 15 to 45% during pregnancy, labor, delivery, or lactation in the absence of any intervention [2].

The World Health Organization (WHO) and UNICEF have approved a comprehensive PMTCT program that includes four key elements: primary HIV prevention among women of childbearing age, prevention of unintended pregnancies among women living with HIV, prevention of HIV transmission from a woman living with HIV to her infant, and provision of appropriate treatment, care, and support to women living with HIV and their children and families [3, 4]. Pregnancy, labor and delivery, and breastfeeding are all times when the virus might spread to the child. It is the primary factor in pediatric HIV infection [2, 5, 6].

Globally, every day 4000 people acquire HIV, including 1100 young people (15 to 24 years old). If current trends continue, 1.2 million people would get HIV for the first time in 2025, which is three times more than the 2025 target of 370,000 new infections [7]. 1.7 million children were living with HIV including 160,000 [110,000- 230,000] new HIV infections among those under five in 2021 but just 52% had access to lifesaving medicines. It means that around half of the children in need of treatment do not have access[8]. In 2021, 70,202 pregnant women were newly infected with HIV and only 81% [63–97%] of HIV-positive pregnant women had access to antiretroviral medications to stop HIV from passing to their children. Despite the remarkable progress in the global HIV response, new HIV infections and AIDS-related deaths remain unacceptably high among pregnant mothers, especially in low and middle-income countries [9].

It is believed that 15 to 45% of children's HIV infections are caused by mothers infecting their newborns during and shortly after birth if no intervention is planned [2]. MTCT rates are still high in Sub-Saharan Africa (SSA) nations, where the great majority of HIV-infected women who are of reproductive age reside. In Ethiopia, MTC transmission prevalence was 9.93% in 2018, according to a systemic review and meta-analysis which is very high. Such high rates continue mostly due to a lack of quality PMTCT services [10].

Poor PMTCT counseling to eligible women, poor providers’ motivation and capacity, inadequate infrastructures, supplies, and equipment, poor provider–client communication, and low client satisfaction were some of the factors identified for the poor quality of option B + PMTCT services [11–13].

The COVID-19 pandemic disrupted health services in low and middle-income countries which in turn affects the health of vulnerable groups such as women and children [14, 15]. In four out of ten countries in Europe and Central Asia, at least half of the women who needed family planning services had significant access issues according to a fast gender assessment conducted by UN Women [16]. It also disrupted services for HIV, sexual and reproductive health, and social protection in eastern and southern African nations with strained and fragile health systems as has been confirmed by non-governmental organizations [7]. Therefore, it is very critical to know the level of the quality of option B + PMTCT services in this era.

In Ethiopia, there are some studies conducted on the quality of option B + prevention of mother-to-child transmission of HIV services however, all of them were conducted before the era of the COVID-19 pandemic [12, 13, 17, 18]. Even though these studies used the Donovedian three-component (structure, process, and outcome) approaches to evaluate the quality of PMTCT services, they omitted several components in each category, i.e. the variables used to measure the structure, process, and outcome dimension of quality are insufficient. Therefore, the current study closed this gap by including all necessary elements. Hence, the current study aimed to determine the level of quality of option B + PMTCT services in public hospitals of the Gamo zone.

## Methods and materials

### Study area and period

The study was conducted at public hospitals in Gamo zone, southern Ethiopia. Gamo zone is found in SNNP regional state and its capital city is Arba Minch which is located 275 km south of the capital city of the southern regional state, Hawassa, and around 505 km southwest of the capital city of Ethiopia, Addis Ababa. According to the data obtained from the zonal health department, the 2019/2020 projected population of the zone was around 1,544,677. As the health department of the zone reported, from the total population 53,449 were estimated to be pregnant. There are 1 general hospital, 5 primary hospitals, 56 health centers, and 302 health posts with 2 health extension workers in each Kebeles (small administrative unit) in the Gamo zone.

### Study design

Facility based cross-sectional study design employing both quantitative and qualitative methods was used.

### Source population

All public hospitals in the Gamo zone were the source population.

### Study population

*All first ANC attendants, delivery ward attendants, PMTCT unit attendants and PMTCT services providers at each hospital were study population. Inclusion and exclusion criteria*

*All first ANC attendants, delivery ward attendants, PMTCT unit attendants and PMTCT services providers at each hospital were included however; those with critical illness at the time of data collection were* excluded.

### Sample size determination

#### Sample size determination for the quantitative data

The sample size for the quantitative part was determined using the single population proportion formula. The sample size calculation depends on the following assumptions: level of confidence (95%), the margin of error (5%), and the proportion of client's satisfaction with PMTCT which is 47% from the study conducted in the Amahara region [19]. The actual sample size for the study was computed using the single population proportion formula as follows.

$$\mathrm n=\frac{{(\mathrm Z\;\mathrm\alpha/2)}^2\;\mathrm p\;(1-\mathrm p)}{\mathrm d2}$$

where, n = sample size, Z_α/2_ = Critical value = 1.96, P = client’s satisfaction by PMTCT service, d = precision (margin of error) = 0.05,

$$\mathrm{Then}\;\mathrm n=\frac{\left(1.96\right)^2\;0.47\left(0.53\right)}{\left(0.05\right)^2}\;=383$$

Considering a 10% of non-response rate the final sample size was *n* = 422.

#### Sample size determination and procedures for qualitative data

##### Observation

Non-participatory direct observations were conducted to assess the compliance of healthcare providers with national PMTCT guidelines. The observation sample size was determined based on the UNAIDS tool for HIV testing and counseling recommendation. A total of 28 sessions were used for direct observation (i.e. seven sessions in each hospital) [2].

##### Record review

A one-month record of ANC, PMTCT, and delivery registers was reviewed.

##### In-depth interviews

It was conducted by MNCH case team leader, clients, and the mother's support group. The purpose of conducting an in-depth interview was to gain in-depth information regarding service management and barriers to PMTCT service implementation. A total of 12 key informants were interviewed. One MNCH case team leader, one purposively selected client and mother’s support group from each hospital were used for in depth interview.

##### Assessment of resources

This was done to determine whether the bare minimum of resources, such as personnel, facilities, logistics, and supplies, were there. The resource assessment tool includes the following items: health care workers, logistics and supplies, the presence of a waiting area, a monthly reporting format, the ANC-PMTCT admission register, the Labor and Delivery Register, the ANC-PMTCT appointment card, gloves, aprons, autoclaves, goggles, sharp boxes, the PMTCT guideline, the PMTCT performance standard, client education materials like brochures, leaflets, and the PMTCT cue card.

#### Sampling technique/procedure for client satisfaction

The study covered all Gamo zone public hospitals offering option B + PMTCT services. In order to determine the appropriate sample size for each hospital, the average number of first ANC, delivery ward, and PMTCT unit attendants at each hospital over the three months prior to the study period were taken into account. After proportional allocation 130,110, 92 and 90 participants were selected consecutive manner until the sample size was satisfied from Arba Minch General Hospital, Chencha Primary hospital, Selam bar primary hospital and Dilfana primary Hospital respectively.

### Data collection tool and technique

A questionnaire for exit interviews was developed after reviewing different works of literature [12, 13, 18, 20]. An observation tool checklist, semi-structured in-depth interview guide, resource inventory tool, and document review template were developed by referring to the national PMTCT guideline of Ethiopia [4].

All of the data collectors started data collection on the same day from the 1^st^ attending mothers and continue until the sample size required for each hospital was obtained. Face-to-face exit interviewer-administered questionnaires were employed to collect the data from mothers. For face-to-face interviews, four BSc nurses participated and for direct observation two trained health officers were recruited. All of the data collectors were selected from outside of the study health facilities to minimize interviewer bias. Furthermore, 2 public health professionals were assigned as a supervisor for the hospitals. An in-depth interview, record review, and resource inventory were conducted by the principal investigator. Each in-depth interview was tape-recorded and transcribed on the same day of the interview sessions.

### Study variable

The independent variables were socio-demographic factors like: residence, age, marital status, educational status, and occupation as well as other factors like travel time, the opening hour of the ANC and PMTCT unit, the amount of time spent with the care provider (counseling time), and the amount of waiting time once you arrive. Input The dependent variable was the quality of option B + PMTCT services.

### Operational definition

#### Input quality dimension

This dimension was evaluated in order to determine whether the people, resources, materials, medications, equipment, and supplies required to provide option B + PMTCT services were available or not**.** Finally, those hospitals having minimum of 75% of resources were categorized as having good quality in the input dimension.

#### Process quality dimension

This dimension was used to reflect how service providers adhere to service standards during a service provision of option B + PMTCT service in the MNCH unit. Service provision processes were observed for seven counseling sessions in each hospital. 33 items were used to assess the quality of care in this dimension. Those hospitals scoring greater than or equal to 90% in more than or equal to four observation out of seven observation was categorized as having good quality.

#### Output dimension

Used to evaluate the ultimate service result of Option B + PMTCT service and patient satisfaction level. Twelve items were used to assess the quality of care in this Donobedian dimension. Hospitals were categorized as having good quality in output dimension if they scored more than or equal to 90% in the assessment for output quality.

#### Overall quality

This particular dimension was determined by combining predetermined three quality components; input, process, and output. Finally hospitals were categorized as having good overall quality if they scored more than or equal 75% in input component and greater or equal to 90% in each components of donobedian model.

#### Client satisfaction

In this context, it relates to how customers view themselves in terms of the accessibility of services and resources, the quality of the service they receive during client-provider interactions, and the flexibility of the service delivery model. A 15-item questionnaire was used to assess customer satisfaction. This survey used a 5-point Likert scale, where 5 represent being extremely satisfied and 1 represents being extremely unhappy. The average of the satisfaction items was used to obtain the mean satisfaction score for each client. An indication of a client's apparent unhappiness was a mean score of 3 or less. Because customers could be reluctant to express their discontent with the services they were receiving, a score of 3 (neutral) was interpreted as indicating dissatisfaction.

#### Waiting time

Is the time clients have to wait before receiving their services.

#### Compliance

In this context, it refers to how well medical professionals adhere to national PMTCT implementation guidelines when conducting tests, offering to counsel, and diagnosing, treating, and documenting patient information.

#### Availability

It refers to the presence of resources such as infrastructure, logistics, and supplies as well as human resources for the implementation of the program by the national guidelines.

#### Drug availability

If a medicine was available when needed during the previous 30 consecutive days, or during the time that data was collected, it is considered to be available in hospitals.

#### Resources

In this context, it refers to the trained health worker, infrastructure, logistics, and supplies (test kits, drugs, IP supplies, FP supplies, HMIS, and job aids).

#### Adherence

Implies that the customer accepts and correctly adheres to the recommended treatment. Both medication adherence and clinical adherence to care are possible.

### Data quality assurance

Various actions were taken to maintain data quality assurance. Data collectors and supervisors received a one-day training session on the protocol to follow before data collection, overviews of the quality of option B + PMTCT services, each part of the tool, and ethical considerations. Every day, the principal investigator or supervisor examined the collected data to ensure its completeness and accuracy. For the benefit of the data collectors and respondents, the Amharic translation of the questionnaire's English version was made. To ensure uniformity, the questionnaire was then translated back into English. With 5% of the overall sample size, the data collecting tool was pretested at Dubo primary hospital. After the pretest, any flaws in the design of the research tool were discovered and strengthened in terms of clarity, understandability, and ease of use for gathering the data needed for the study.

### Data processing and analysis

Quantitative data were checked for its completeness, edited, cleaned, coded, and entered into Epi data version 3.1 and exported to IBM SPSS version 25.0 for analysis. Univariate data analysis was conducted to estimate the prevalence of variables for respective quality components. Results were presented by tables and graphs. Qualitative data was analyzed manually using the content thematic approach. This involved reading the script several times and translating transcripts from the local language to English. The main study themes were fitted with three quality components, whilst categories were barriers for poor service quality in each identified theme. Finally, it was presented with the quantitative result through triangulation. Direct quotations were presented reflecting reasons for poor service quality.

## Results

### Health facilities characteristics

This study was conducted in public hospitals which are found in the Gamo zone, southern Ethiopia. Among them, Arba Minch general hospital and Dilfana primary hospital are found in Arba Minch town. Other hospitals are Garase primary hospital, Kamba primary hospital, Selam Bar primary hospital, and Chencha primary hospital which are found in Garase district, Kamba district, Kucha district, and Chencha Districts respectively. Since two of these hospitals such as Garase primary hospital and Kamba primary hospital have no PMTCT or ART units, mothers who tested positive for HIV while pregnant, giving birth, or during lactation were directed to the nearby health centers. As a result of the above reason, this study was conducted in four hospitals other than the above two hospitals.

### Characteristics of study participants of exit interview

Of the 422 participants selected for the exit interview, 386 of them responded giving a response rate of 91.90%. From these, 238(61.7%) of the study participants were from urban areas and the mean age of study participants was 26.8(SD ± 4.69). Most of the study participants 377(97.7%) were married. It was revealed that 277(71.8%) of the study participants of exit interview knew about PMTCT services and 120(43.3%) of them got information about PMTCT from health workers. The majority of the participants 353(91.5%) responded that the opening hour of the MNCH clinic was convenient for them. For more than half of the respondents 234(60.6%), it took more than 30 min to arrive at the health facility and it also took more than 30 min to receive services for around half of the respondents 186(48.2%) (Table 1).

**Table 1:** Characteristics of study participants for exit interview, Ethiopia, 2022 (*n* = 386)

Variables	Categories	Frequency	Percent%
Age	20 – 29	263	68.1
	30—39	121	31.3
	≥ 40	2	.5
Level of education	Illiterate	39	10.1
	Read and write	72	18
	Elementary school (1–8)	47	12.2
	9 -12	134	34.7
	Diploma and above	94	24.4
Residence	Urban	238	61.7
	Rural	148	38.3
Informed about PMTCT before	Yes	277	71.8
	No	109	28.2
Source of information about PMTCT	Health workers	120	43.3
	Mass media	29	10.4
	Friends	80	28.9
	Health extension workers	48	17.4
Convenience of opening hour of the clinic	Yes	353	91.5
	No	33	8.5
How much time it take to arrive at hospital	< 30 min	234	60.6
	> 30 min	152	39.4
How much time it takes to receive services	< 30 min	200	51.8
	> 30 min	186	48.2

### Input quality dimension

#### Human resource and infrastructure

In this study, only one hospital had service providers who had received PMTCT training per standards (more than or equal to 6 trained providers on option B + PMTCT services in each hospital). Out of four hospitals, two (or 50%) have waiting areas and counseling rooms with good ventilation. Even though the water was accessible in the form of pipe water in all hospitals, only one hospital had hand hygiene facilities in the MNCH unit including the PMTCT room. In general, only one hospital out of the four hospitals has all the infrastructure and human resource inputs (Table 2).

**Table 2:** Input PMTCT service quality performance verification indicators in hospitals of Gamo zone, Southern Ethiopia

Input items	Number of hospitals responded “ yes”	Percent (%)
**Human resource and infrastructure**		
Trained service providers on option B + PMTCT services per standard in MNCH clinic	1	25.0
Well ventilated waiting room	2	50.0
Wel Ventilated counseling room	2	50.0
Hand washing facility availabilities in the MNCH unit including PMTCT room	1	25.0
**Medical supplies and Lab tests**		
HIV rapid test	4	100.0
Supplies required for CD4 sample transport	4	100.0
DBS Kit	2	75.0
Pregnancy test	4	100.0
Viral load test	0	0
CD4 testing Machine	0	0
VDRL Test	4	75.0
CBC Test	4	100.0
ARV drugs including suspension for pediatrics	4	100.0
OI drugs like cotri,	2	100.0
Glove all types	2	50.0
Goggles	2	50.0
Autoclave	2	50.0
**Job aids, Patient forms and registers**		
Technical guide line/option B + referral manual	4	100
PMTCT broachers and leaflets	3	75
All HMIS forms and registers available	4	100
All lab request forms	2	50
ANC/PMTCT appointment card	4	100
Counseling and testing protocol	3	75
All PMTCT cohort tools	3	75
HFs with all patient forms and registers	2	50

#### Medical supplies and Lab tests

The results of this investigation showed that while DBS kits were only available in two hospitals, all hospitals had access to HIV rapid tests and the supplies needed to transport CD4 samples. All of the hospitals had VDRL tests, pregnancy tests, and CBC tests, but none of them had viral load tests and CD4 testing machines (Table 2).

#### Job aids and IEC materials

Only two hospitals had access to all lab request forms, whereas all HMIS forms and registers were available in all hospitals. They all had access to the referral handbook or guidelines for option B + PMTCT services. Three of the institutions had PMTCT broachers and leaflets, but all of them had ANC/PMTCT appointment cards. In three hospitals, testing and counseling services were available (Table 2).

#### Process quality dimension

According to the finding of this study, healthcare providers introduced themselves to clients and discussed the need and benefit of HIV testing in three of the health facilities however; they explained the HIV testing procedure and the possible HIV test results in two of them. Waiting time was a standard in half of the hospitals. It had been observed that HCPs informed the clients when the results were ready and how and where to receive the results in three hospitals but they explained procedures to safeguard the confidentiality and the need for shared confidentiality in two of the health facilities. Regarding disclosure issues, clients were reminded that their result does not indicate their partner's HIV status and encouraged testing if not and addressed disclosure issues in three hospitals. It had been also observed that HIV-positive clients were greeted with respect and called by their names in all of the hospitals. In addition, the possible barriers to adherence such as stigma and living situation were reviewed in two hospitals. Furthermore, screening for opportunistic infections, STIs and TB were done in three hospitals but screening for cervical cancer was done in only one hospital (Table 3).

**Table 3:** Process PMTCT service quality performance verification indicators in hospitals of Gamo zone, southern Ethiopia

Process quality items	Number of facilities responded ‘yes’	Percent (%)
**Observation sessions at Anti-Natal Care service unit**		
Waiting time is as a standard	2	50
Greeting of clients with respect	4	100
HCPs introduce themselves to clients	4	100
HCP discuss the need and benefits of HIV testing	3	75
HCP explain HIV testing procedure	2	50
HCP explain the possible HIV test result	2	50
HCP inform the client when the result will be ready and how and where to receive the result	3	75
HCP explain procedures to safe guard confidentiality and the need for shared confidentiality	2	50
HCP provide result clearly and simply	3	75
HCP review meaning of the result, including window period for the negative tests	2	50
HCP reinforce the need to consider the test result in reference to most recent risk exposure	2	50
HCP use language that client can understand	4	100
HCP reinforce prevention messages (A, B, C) so that patient can stay negative	3	75
HCP remind the client that her result does not indicate partner’s HIV status and encourage to test if not	3	75
HCP clearly explain the next appointment date and available services at the health facility	3	75
HCP record all information related to service	4	100
**Observation sessions at PMTCT unit**		
HCP greet client with respect	4	100
HCP introduce him/ herself for client	2	50
HCP call client by name	2	50
HCP discuss current health status with client including overall health and current problems; the latest laboratory result including CD4	2	50
Conduct physical examination	4	100
HCP review with client possible barriers to adherence; stigma, living situation, travel to clinic for refill of medication, side effect, depression etc	2	50
HCP make screening for opportunistic infections	3	75
HCP make screening for STI	3	75
HCP make screening for cervical cancer	3	75
HCP review understanding of the client including: asking client to describe her ARV regimen and how to take medications	4	100
HCP discuss about the need of partner notification	2	50
HCP introduce about safe sex practice	2	50
Discuss about nutritional support	2	50

#### Output quality dimension

**Fig. 1:**
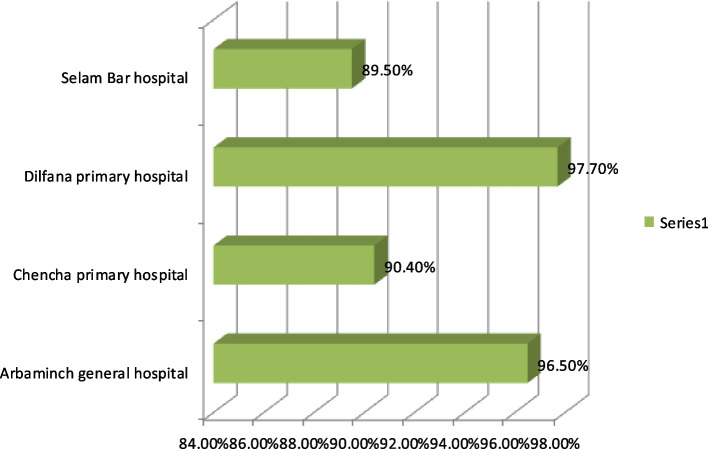
Level of satisfaction of clients toward option B + PMTCT services in the hospitals of Gamo Zone, Southern Ethiopia

According to this study's findings, two hospitals' PMTCT services satisfied more than 95% of their patients (Fig. 1). All pregnant, laboring, and lactating mothers received HIV testing and counseling in all hospitals, and those who tested positive for HIV were given ART per the national target (> 95 percent). Additionally, it was shown that only half of the hospitals had achieved the target (> 90) for client treatment adherence and partner HIV testing. Confirmatory anti-body testing was carried out per the national target however; the average turnaround time for DBS results was longer than two weeks in all hospitals, which is not what is expected across all hospitals. As the study finding showed all hospitals administered cotrimoxazole and ARV prophylaxis to newborns that were HIV-positive. Pediatric patients who tested positive for HIV were almost always enrolled in chronic HIV care. Initial and subsequent visits' CD4 counts were monitored in accordance with the national target (> 90%) in two hospitals (Table 4).

**Table 4:** Output service quality performance verification indicators in hospitals of Gamo zone, Southern Ethiopia

Output variables	Number of Hospitals	Percent
Client satisfaction per the standard (> 95%)	2	50.0
Performed HIV testing and counseling for pregnant,laboring and lactating mothers as per national target	4	100
HIV positive clients (pregnant, laboring and lactating) received ART as per national target (> 95%)	4	100
Performed partner testing as per national target (> 90%)	2	50.0
Monitor CD4 count at initial and follow up visit per national target (> 90%)	2	50.0
Average waiting time spent to see the counselor per standard (> 95%)	2	50.0
Enrolled HIV reactive pediatrics to chronic HIV care per national target (> 95%)	4	100
Average DBS result turnaround time per the standard ( within two weeks)	0	0
Conduct confirmatory anti-body test per national target (> 90%)	4	100
HEIs received cotri-moxazole as per national target	4	100
ARV prophylaxis received as per national target	4	100
Clients with good treatment adherence level per standard (> 90%)	2	100

### The overall quality of PMTCT services

In this study, no hospitals simultaneously met the requirements for good quality in all three dimensions of option B + PMTCT service quality. Only one hospital out of the four hospitals met the requirements for good quality of option B + PMTCT service in the input dimension. Regarding the process and output dimension's quality of PMTCT services, two of the hospitals met the criteria for good quality. One hospital out of the total exhibited poor performance in all three areas of service quality for PMTCT.

### Contributing factors/Barriers for poor quality of PMTCT services

#### Barriers for the input dimension's poor quality of PMTCT services

Healthcare providers identified the following input-related aspects as hurdles to the quality of Option B + PMTCT service: various infrastructure, resource, or supply chain issues, and a lack of a qualified human workforce as described below:

*"……There are no hand washing facilities in the MNCH department, even though we are aware that good hand hygiene practice is essential to the prevention and control of the transmission of infection. To address this issue, hospital managers must collaborate with other interested parties."(PMTCT service provider* = *1).*

*"…There are no enough staff members trained in option B*+*PMTCT services. Even though the criteria for PMTCT service provision suggests at least six trained health professionals on option B*+ *PMTCT service in each hospital, there are currently just two healthcare professionals at this facility who are trained in this service. Therefore how will PMTCT service quality be enhanced in the absence of sufficient trained manpower?" (PMTCT service provider*=*2)*

*"…..In this unit, there is a lack of many consumables, including DBS sample collection kits and OI medications such as co-trimoxazole and prednisolone. Additionally, it is challenging to offer high-quality PMTCT services due to the lack of a viral load and CD4 testing machine in hospitals in the Gamo zone.”(PMTCT service provider*=*3).*

#### Barriers for the process dimension's poor quality of PMTCT services

Most of the respondents or clients agreed that there is no problem during the provision of PMTCT service however; some of the PMTCT service users forwarded their suggestion on the process of PMTCT service provision as follows:

*"……We are suffering excessive waiting times to receive the service even though the respect and secrecy of health professionals in this room are commendable, which is consistent with the quantitative finding reported in *Table 2*" (one of PMTCT service users).*

*‘’…… Since they will be overworked and consequently make clients wait a long time to receive PMTCT services, it is preferable if the trained healthcare professionals are only assigned to the PMTCT unit. Additionally, we cannot find a trained health professional to give the services when the trained PMTCT providers are assigned to the on-duty program'' (PMTCT service provider*=*4).*

#### Barriers for the output dimension's poor quality of PMTCT services

The biggest issue according to the key informants is the prolonged DBS turnaround time. It is also difficult to monitor CD4 count at follow-up visits because there is no CD4 machine at surrounding hospitals. Additionally, they said that there is no close supervision and monitoring of the PMTCT services by the responsible bodies.

*‘’…..Since we only hold meetings once a month before reporting the monthly report, there is insufficient ongoing supervision, monitoring, and review of the performance of the MNCH, including the PMTCT unit. As a result, the aforementioned issue needs to be resolved to improve the quality of PMTCT services’’ PMCT service provider*=*5.*

*"…..Despite the PMCT guideline's recommendation that CD4 counts be taken at the initial and follow-up visits, it is impossible to determine the clients' CD4 counts due to the lack of CD4 machines in the hospitals nearby therefore it takes much time until the result returned to the facility from the regional laboratory and the DBS turnaround time is also over a month". PMTCT provider*=*6.*

## Discussion

This facility-based cross-sectional study attempted to determine the level of quality of option B + PMTCT services at public hospitals in the Gamo zone, southern Ethiopia. It also explored the contributing factors of poor quality of PMTCT services in the study facilities. Donobedian model consisting of three components such as input, process, and output components was used to evaluate the level of quality of option B + PMTCT services [21].

The findings of this study have shown that no hospitals simultaneously met the requirements for good quality in all three dimensions of option B + PMTCT service quality. Out of the four hospitals, only one met the standards for good quality option B + PMTCT service in the input dimension. However; two of the hospitals (50%) satisfied the requirements for good quality in option B + PMTCT services in terms of process and output dimension. Also from the total number of hospitals, one performed poorly in each of the three service quality components for option B + PMTCT services**.**

This result was below from the research findings conducted in the Mekelle zone, northern Ethiopia. According to the above study, the overall level of service quality of Option B + PMTCT service was rendered as good in one out of six(16.7%) of the studied health facilities. In particular, 33.3 percent of health institutions reported good input service quality, but only 25 percent of them also reported good process and output service quality [17]. The limited sample size in our study may have contributed to this discrepancy because only four hospitals were studied in our study to evaluate the quality of option B + PMTCT services. The COVID-19 expansion, which may have an impact on the accessibility of different supplies and equipment in healthcare facilities, could be another factor since this study was conducted during the era of COVID-19 expansion.

Regarding the contributing factors of quality of option B + PMTCT services different factors were explored by in-depth interview. In the qualitative findings, one of the PMTCT service providers explained that the main contributing factor for the poor quality of option B + PMTCT services was *“lack of hand washing facilities in the MNCH department including PMTCT room.”* Another health care provider complained that “*There are no enough staff members trained in option B* + *PMTCT services therefore how PMTCT service quality will be enhanced in the absence of sufficient trained manpower?"* This could be due to the fact that providing option B + PMTCT services need adequate trained health care providers and basic infrastructure like hand washing facilities. This result was similar with the study findings conducted in public hospitals of Hadiya Zone, Southern Ethiopia, Gamo Zone, Southern Ethiopia and Gondar, Northern Ethiopia. This finding was also similar with the study conducted in Ghana [11, 13, 22, 23].

According to another qualitative findings of this study, one of the PMTCT service users explained that the main contributing factor to the poor quality of option B + PMTCT services was *"……We are suffering excessive waiting times to receive the service even though the respect and secrecy of health professionals in this room are admirable”.* One of the health care providers also complained that *‘’… Since it is obvious that health care providers overloaded and consequently make clients wait a long time to receive PMTCT services, it is preferable if the trained healthcare professionals are only assigned to the PMTCT unit.* The possible explanation for this may be waiting long time to receive services may affect satisfaction which may in turn affect client adherence with the service in the future. This study finding was similar with the study conducted in Gamo Zone and Hadiya Zone, Southern Ethiopia. It was also similar with the study conducted in Nigeria [11, 13, 24].

Furthermore, the qualitative finding of this study showed that the quality of PMTCT services was affected by the different factors.. These finding was explained by two health care providers as follows *‘’…..Since we only hold meetings once a month before reporting the monthly report, there is insufficient ongoing supervision, monitoring, and review of the performance of the MNCH, including the PMTCT unit”* as explained by one of the HCP*.* Another HCP also explained that *"…..Even though the PMCT guideline recommends that CD4 counts be taken at the initial and follow-up visits, it is impossible to determine the clients' CD4 counts due to the lack of CD4 machines in the nearby hospitals".* This could be due to the fact that ongoing supervision and monitoring improve services quality if it is done countinously. This study finding is similar with the study done in Northern Ethiopia, Tigray and Amhara Region [17, 18].

### Limitation of this study

There were limited studies that could be used to compare the findings of this study because other studies conducted on this specific topic concentrated on determining the level of quality of option B + PMTCT services by only measuring client satisfaction. In contrast, our study included client satisfaction as one of the components of the output dimension of the Donobedian model [12, 13, 18]. No regression model was applied in this study because this study used Donobedian model to determine the level of quality in the service provision of option B + PMTCT services.

## Conclusion

According to established quality evaluation criteria for each quality component, no healthcare institutions/hospitals achieved the intended level of quality. That means no hospitals simultaneously met the requirements for good quality in all three dimensions of option B + PMTCT service quality. However; two of the hospitals (50%) satisfied the requirements for good quality in option B + PMTCT services in terms of process and output dimension. Having no ongoing supervision, monitoring, and review of the performance of the PMTCT unit, inadequate trained HCPs on option B + PMTCT services, prolonged DBS turnaround time, resource limitations/constraints like an absence of CD4 machines in nearby hospitals, assigning trained HCPs in different units in addition to PMTCT unit, and excessive waiting time were barriers affecting the quality of option B + PMTCT services negatively. PMTCT unit performance must be continuously monitored, reviewed, and supervised. To obtain the minimum required resources to improve the quality of PMTCT services primary hospitals must be supported.

## Data Availability

The datasets used and/or analyzed during the current study are available from the corresponding author upon reasonable request.
